# Supplementary material for: Hybrid Models and Biological Model Reduction with PyDSTool
Source: PLoS Comput Biol. 2012 Aug 9;8(8):e1002628. doi: 10.1371/journal.pcbi.1002628 (PMC3415397; doi:10.1371/journal.pcbi.1002628)
Supplement: Text S4 — Complete source code for the PyDSTool package (version 0.88.120504). Includes API documentation and help files linking to web pages. This file is identical to the current public release on Sourceforge.net. (ZIP) [file pcbi.1002628.s004.zip › PyDSTool/html/toc-PyDSTool.Toolbox.optimizers.helpers-module.html]

xml version="1.0" encoding="ascii"?


helpers


# Module helpers

---

## Classes

CenteredFiniteDifferences  
 FiniteDifferencesFunction  
 ForwardFiniteDifferences  
 ForwardFiniteDifferencesCache  
 Quadratic  

## Variables

helpers\_\_all\_\_

---

[hide private]
